# Attacks depriving people of urgently needed health care

**DOI:** 10.2471/BLT.17.020117

**Published:** 2017-01-01

## Abstract

Targeting health care has become a deliberate military strategy. How can the international community make conflict parties respect the rules of war? Jan Dirk Herbermann and Fiona Fleck report.

The Sudan Liberation Movement forces were edging closer from one side and the Sudanese army was moving in from the other side.

“The health-care centre was literally on the frontline,” recalls Ali Naraghi, who was working for the International Committee of the Red Cross (ICRC) in Sudan’s Darfur region in 2005, when the clashes took place.

Naraghi recalls how the health-care centre was hit so many times by mortar, shells and rockets that it ceased to function. “Nobody could visit the health-care centre anymore. The humanitarian consequences for people living in the region were catastrophic.”

Today Naraghi heads Health Care in Danger, a project initiated by the International Red Cross and Red Crescent Movement.

Naraghi and his team are working to improve the security of health-care facilities and workers in conflicts and other emergencies, and to ensure that these facilities remain accessible to people in need.

Last year, the eastern part of Aleppo in the Syrian Arab Republic was left without any functioning hospitals, as the battle to control the city intensified. On 21 November, Elizabeth Hoff, WHO Representative in the country, briefed the UN Security Council on the health situation in Syria, via video link. She described the dire health situation inside the country, the challenges it presents for WHO’s work on the ground and appealed to the Council “to use every last ounce of influence” to end the attacks on health facilities there.

Some attacks on health care are widely reported by the media, others receive less attention.

“Not only in the Syrian Arab Republic, Afghanistan and Yemen – these attacks occur in Libya, South Sudan, the Central African Republic, Nigeria, Somalia and other countries,” says Naraghi. “Every attack on health care is a tragedy, every attack is an attack on humanity.”

“Every attack on health care is a tragedy, every attack is an attack on humanity.”

Ali Naraghi

The *Report on attacks on health care in emergencies* released last year by the World Health Organization (WHO) recorded a total of 594 attacks on health-care facilities between January 2014 and December 2015, resulting in 959 deaths and 1561 injuries in 19 countries facing emergencies. Of those 594 attacks, 62% (366) were reported as deliberate.

The WHO report – an attempt to shed light on the magnitude of this phenomenon – is based on consolidated and secondary data from 2014 and 2015 and is, therefore, not comprehensive due to the challenges of gathering data in emergencies.

According to the report, health-care workers were killed, kidnapped or assaulted, health facilities were damaged, destroyed or taken over for non-medical purposes and ambulances were looted, stolen and shot at.

For Erin Kenney, a technical officer from WHO’s Health Emergencies Programme, such attacks not only stop health services getting to those in need, but threaten long-term public health goals including progress towards universal coverage of health services.

“Each health facility bombed, each medical worker killed is a tragic loss that means years of investment destroyed,” says Kenney, adding: “WHO is finalizing its data collection method on such attacks, so that we can have more authoritative information to focus our collective efforts to stop these attacks.”

Last year, the United Nations Security Council condemned attacks on health workers and facilities in conflict situations and demanded an end “to impunity for those responsible and respect for international law on the part of all warring parties” in Resolution 2286.

Although there are no data to allow a clear comparison, some emergency health experts believe that deliberate attacks on health care have been on the rise in recent years.

“When you look at what happens in the Syrian Arab Republic and Yemen, hospitals are being targeted more than they used to be,” says Professor Paul Spiegel, director of the Johns Hopkins Center for Humanitarian Health in the United States of America (USA).

“In the past, the symbol of the Red Cross and Red Crescent Movement was a sign of protection, now it’s a more of a symbol to bomb,” says Spiegel, who was deputy director of the Division of Programme Support and Management at the United Nations Refugee Agency until last July.

Strikes on health-care targets have dramatic short- and long-term impacts.

“It’s not only the targeted hospital that is damaged or destroyed and the people killed and injured,” says Bruno Jochum, Director of Médecins Sans Frontières (MSF) Switzerland.

“The future of all health-care delivery in conflict countries is at stake,” Jochum says, citing the Kunduz incident as an example. In October 2015, USA forces bombed and destroyed the MSF trauma clinic in the city in northern Afghanistan.

“The future of all health-care delivery in conflict countries is at stake.”

Bruno Jochum

“We have still not restarted our activities in the Kunduz region because we haven’t received security assurances from the conflict parties,” he says. The people in the Kunduz region pay a heavy price: more than one million men, women and children have no access to surgical care. “On average we had about 15 000 surgeries a year,” Jochum says. “Without our support, many people in the Kunduz region will die.”

MSF says that the clinic was deliberately hit by the USA forces and argues that the incident could amount to a war crime, but the USA government rejects the charges, citing a USA Department of Defense investigation that found that the incident was due to human error and equipment failures.

Jochum critizes the USA investigation as “flawed, since it was not independent,” but concedes that “at least the USA military has acknowledged its responsibility and taken some internal measures”. In many other conflicts, governments not only deny their responsibility for attacks on health care, but do not even investigate them.

“When governments wage a war on terrorism they tend to disrespect international humanitarian law and argue that the provision of impartial medical aid is the same as providing assistance to the enemy,” Jochum says.

“Governments label the enemy as ‘terrorists’, the enemy is thus criminalized and – in their view – loses the right to protection under international humanitarian law, especially the right of wounded combatants to medical treatment, which is one of the founding blocks of the Geneva Conventions,” he says.

Jochum and other experts agree that deliberate military attacks on health-care facilities aim to demoralize populations and deprive the enemy of medical care.

For Tobias Vestner, Cluster Leader within the Security and Law Programme of the Geneva Centre for Security Policy, an international foundation, strikes on health targets in rebel-held areas are part of the military strategy of certain groups: “The main goal is to terrorize and punish people who are suspected of cooperating with the opposition forces”.

For Vestner, who was a member of the Swiss government delegation at the negotiations on the 2014 Arms Trade Treaty, attacks on health care make a mockery of international law and the institutions that uphold it.

He believes that the current legal framework to protect health-care facilities is adequate: “The Geneva Conventions and additional protocols prohibit attacks on health-care facilities, unless they are used to commit acts that are harmful to the enemy, such as using them to launch attacks. Protection from attacks may cease only after due warning has been given and after that warning goes unheeded.”

The Conventions are reinforced by the Rome Statute, the treaty that established the International Criminal Court in 1998, that includes in its list of war crimes “intentionally directing attacks against … hospitals and places where the sick and wounded are collected, provided they are not military objectives”. 

So how can conflict parties be persuaded to stop attacking health-care targets?

Marking facilities more clearly has traditionally been the main way to protect health facilities, but this only works when targeting is accidental and therefore new strategies are needed to ward off deliberate attacks.

Working with their partners, the Health Care in Danger team has identified a set of measures to prevent and mitigate attacks on health care.

“Now these measures need to be translated into action,” says Naraghi, adding that the main hope for stopping the attacks is through diplomatic efforts, especially confidential dialogue with the warring parties.

Other measures to protect health-care facilities and other civilian targets include establishing no-fly zones for military aircraft in countries in conflict. This was effective in Libya in 2011, Vestner notes, when the Arab League called on the United Nations to set up a no-fly zone over the country to protect civilians.

Economic sanctions, including travel restrictions, are another possibility, Vestner adds, but it is often a challenge to win political support for such action in the United Nations Security Council.

“We are identifying measures that have demonstrated effectiveness in reducing the risk of attacks or building resilience of health-care facilities and workers after attacks,” says Kenney.

“Equipped with more complete data and effective tools to tackle the problem, we hope that we can make more progress in stopping such attacks from taking place in future.”

**Figure:**
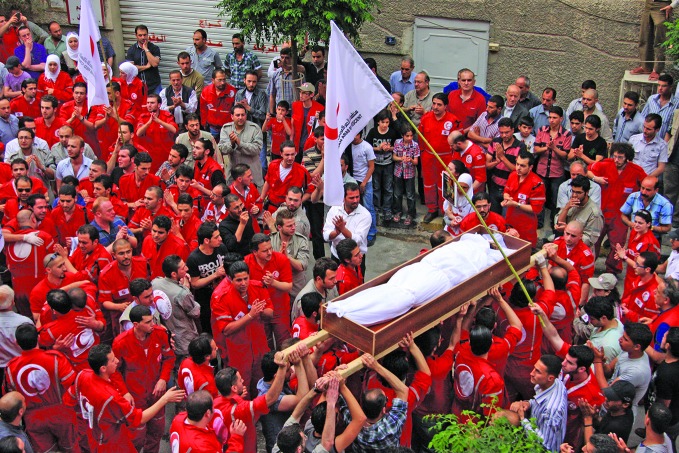
Funeral in the Syrian city of Douma of Mohammad al-Khadraa, a Syrian Arab Red Crescent first-aid responder, who was killed on duty on 24 April 2012. He was shot dead in a vehicle clearly marked with the Red Crescent emblem.

ICRC: Ibrahim Malla

**Figure:**
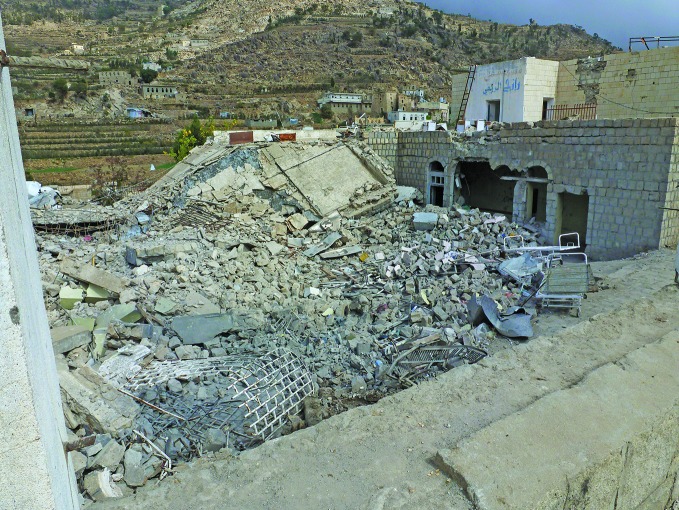
Shiara Hospital, an MSF-supported facility in the Razeh district of northern Yemen, after being hit by a projectile on January 2016.

MSF

